# Commentary on “The road to reliable peptide assays is paved with good guidelines”

**DOI:** 10.1111/cen.14894

**Published:** 2023-03-13

**Authors:** Anna M. Kowalka, Kleopatra Alexiadou, Joyceline Cuenco, Rosemary E. Clarke, Stephane Camuzeaux, James Minnion, Emma L. Williams, Paul Bech, Sanjay Purkayastha, Ahmed R. Ahmed, Zoltan Takats, Bernard Khoo, Harry J. Whitwell, Maria G. Romero, Stephen R. Bloom, Matthew R. Lewis, Tricia M.‐M. Tan

**Affiliations:** ^1^ Section of Diabetes, Endocrinology and Metabolism, Department of Metabolism, Digestion and Reproduction Imperial College London London UK; ^2^ Blood Sciences Department Raigmore Hospital Inverness UK; ^3^ Section of Bioanalytical Chemistry, Department of Metabolism, Digestion and Reproduction Imperial College London London UK; ^4^ National Phenome Centre Imperial College London London UK; ^5^ Department of Clinical Biochemistry, North West London Pathology Charing Cross Hospital London UK; ^6^ Department of Surgery and Cancer Imperial College Healthcare NHS Trust London UK; ^7^ Endocrinology, Division of Medicine University College London London UK

**Keywords:** bariatric surgery, mass spectrometry, peptide YY

We note that Maurer et al., in their correspondence,^1^ have raised some concerns about the preanalytical and analytical validation of the peptide YY (PYY) assay described in our paper ‘The postprandial secretion of peptide YY_1__–36_ and _3__–36_ in obesity is differentially increased after gastric bypass versus sleeve gastrectomy’.^2^ The validation and assay characteristics were not the primary objectives of the paper. We focussed on the physiology of PYY secretion in healthy volunteers and patients pre‐ and post‐gastric bypass versus sleeve gastrectomy. Therefore, we did not include a full description and details of the assay validation procedure and history. We agree with the overall principles that Maurer et al. subscribe to and summarise herewith our findings to show that we have followed and fulfilled internationally accepted standards in the validation of our assay.

We have verified stability of PYY_1__–36_ and PYY_3__–36_ at preanalytical, analytical and post‐analytical stages of the assay. At preanalytical stage the whole blood is collected in prechilled lithium heparin (LH) or ethylenediaminetetraacetic acid (EDTA) tubes and containing evaluated in‐house inhibitors (DPP4 inhibitor and Aprotinin). The sample is centrifuged immediately at 4°C and flash‐frozen on dry ice or transferred immediately to a −80°C freezer. The samples are kept at −80°C until being thawed once only for analysis.

We deemed our protease inhibitors sufficient for maintaining stability of PYY_1__–36_ and PYY_3__–36_ and inhibiting peptide degradation. Our data showed no degradation of PYY_1__–36_ or PYY_3__–36_ for either EDTA or LH plasma at the 3 h time‐point at room temperature (19°C) and the 3 h time‐point at 4°C, with or without inhibitors. The mean concentration from injections of triplicate QCs (low and high) showed that for spiked samples and samples from a healthy volunteer, the concentrations remained similar regardless of the presence of inhibitors or their absence (with the mean percentage difference for overall and between all tested sample sets ≤10%).

Maurer et al. have quoted Toräng et al.'s study on PYY's major cleaving sites but disregarded the outcome from their study of the inhibitory effects of EDTA on metalloendoproteases, neuroendopeptidase (NEP) and Meprin *β*.^3^ Toräng et al. demonstrated that the risk of PYY degradation in collected blood is small in collection tubes containing EDTA. We have studied the stability of both PYY species with our inhibitor mix containing DPP4 inhibitor and Aprotinin, where we spiked PYY_1__–36_ and PYY_3__–36_ into plasma EDTA and LH from three different volunteers. We did not detect any significant differences in concentrations of both peptides with only these inhibitors present suggesting that NEP, Meprin *β* and Am‐P activity is negligible under these conditions.

Furthermore, we have verified the stability of both species of PYY in both EDTA and LH plasma under the following conditions: (1) freezing and thawing up to a second cycle; (2) incubation at 4°C for up to 4 h; (3) long‐term storage (6 months) at −80°C; (4) postextraction, during storage in the autosampler at 4°C. We have verified the stability of our stock and working stock solutions for up to 7 months, stored at 4°C. We would state that it is a matter of standard operating procedure that we store these solutions at −80°C and discard these at the end of the day of the run after thawing, affording a safety margin. Last, our assay has been validated both with plasma‐based calibrants and QCs, and cross‐validated in a surrogate matrix (20BMA). We also validated matrix effects (ME) in spiked plasma EDTA and plasma LH. Mean percentage ME for PYY_1__–36_ was 100.7% and for PYY_3__–36_, 111% in plasma LH. EDTA ME results were reported previously.^2^

Maurer et al. suggest that imputation of <LLOQ (lowest limit of quantitation) values as zero (method M7 as listed in Johnson^4^), as specified in our study, is the ‘worst’ method for handling LLOQ values. The conference paper of Johnson examined the impact of setting <LLOQ values to zero on the estimation of pharmacokinetic values for drugs, utilising a model which is highly dependent on the last few low‐level sample values. Crucially, our study is not a pharmacokinetic study, but instead a physiological study where we are examining the relatively large hormonal responses to a mixed meal particularly after surgery. Hence, Maurer et al.'s criticism seems to be misplaced, as they did not take account of this crucial difference. Moreover, we have carried out a sensitivity analysis of our data where we imputed <LLOQ values with ½ the LLOQ (method M5 as listed in Johnson^4^), and consistent with the method used by Reverter‐Branchat et al.^5^ This method did not alter the contrasts and *p*‐values to a significant degree, suggesting that any bias introduced by the different imputation method for values <LLOQ was small and does not affect the conclusions from our study.

In conclusion, we agree that it is important to carefully follow international analytical guidelines to validate assays. Our assay is indeed validated according to such guidelines and the conclusions made from the data in our paper are indeed valid.

## AUTHOR CONTRIBUTIONS

All authors contributed to the writing of the manuscript and approved the final version of the manuscript. Tricia M.‐M. Tan is the guarantor of the manuscript.

## CONFLICTS OF INTEREST STATEMENT

James Minnion, Stephen R. Bloom, Tricia M.‐M. Tan are employees and/or share‐holders in Zihipp Ltd., an Imperial College spin‐out company developing analogues of gut hormones for treatment of obesity. All other authors declare no conflict of interest.

## Data Availability

Data are available from the corresponding author upon reasonable request.
